# Chitosan Hydrogels Enriched with Biocompounds Extracted from Marine Sponges: Potential to Modulate the Inflammatory Process in an *In Vitro* Study

**DOI:** 10.1021/acsomega.5c01171

**Published:** 2025-06-12

**Authors:** Mirian Bonifacio, Cíntia C. Santi Martignago, Dalete C. S. Souza, Homero Garcia-Motta, Laís C. Souza-Silva, Beatriz Soares-Silva, Karolyne S. J. Sousa, Anabella. P. Rosso, João H. G. Lago, Alessandra M. Ribeiro, Marcelo Assis, Renata N. Granito, Ana Rennó

**Affiliations:** † Department of Biosciences, 28105 Federal University of São Paulo (UNIFESP), Rua Silva Jardim 136, Vila Matias, Santos, SP 11015-020, Brazil; ‡ Center for Natural and Human Sciences, 74362 Federal University of ABC (UFABC), Santo André, SP 09280-560, Brazil

## Abstract

Hydrogels are recognized as effective drug delivery systems in medical and pharmaceutical applications. Among them, chitosan (CH) hydrogels stand out for their biocompatibility, biodegradability, and ability to release bioactive substances. This is especially promising for anti-inflammatory compounds since inflammation is associated with various diseases. Although effective, conventional anti-inflammatory drugs, when administered orally and over the long term, can cause side effects, stimulating the search for natural alternatives and safer release systems. Furthermore, in the search for natural bioactives, it is known that a source of biocompounds that is still little explored, despite its potential, is the marine sponge Dysidea robusta. The aim of this study was to develop and characterize chitosan-based hydrogels enriched with biocompounds derived from the marine sponge Dysidea robusta. Four chitosan hydrogel formulations were synthesized using varying concentrations of urease and urea, and their physical and morphological properties were evaluated using the mass loss test and techniques such as Fourier transform infrared spectroscopy (FTIR), scanning electron microscopy (SEM), and rheology. Seven biocompounds from the marine sponge Dysidea robusta (DR1 to DR7) were obtained and incorporated into a hydrogel formulation with optimal gelation time, stability, and presence of pores (H3). Drug release capacity was analyzed, as well as *in vitro* biological activity through viability and cell proliferation assays with fibroblasts and chondrocytes, along with immunoassays for pro-inflammatory cytokines IL-6 and TNF-α in macrophages. The results showed that all the hydrogels were stable and biocompatible, with H3 being selected due to its physical profile. Notably, the hydrogel enriched with the DR5 compound significantly reduced IL-6 levels and showed potential for controlled drug release over time. These findings highlight the promise of chitosan hydrogels as injectable carriers for natural anti-inflammatory compounds, suggesting their applicability in therapies for inflammatory diseases.

## Graphical abstract

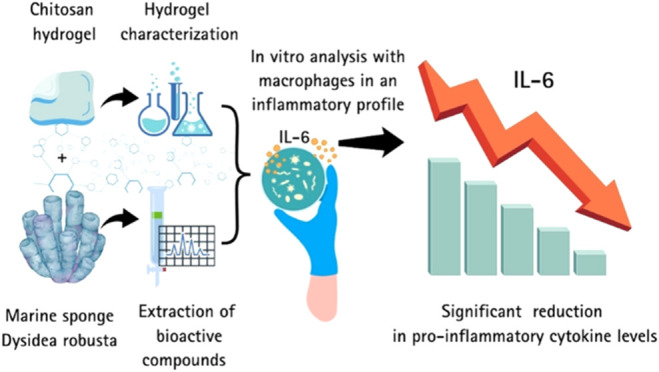

## Introduction

The development of drug delivery systems based on polymeric matrices has attracted the attention in the pharmaceutical field, especially using natural matrices.^1,2^ Natural polymers present biodegradability, biocompatibility, is easy to obtain and have positive effects on cell proliferation.^3^ One of the most effective natural polymers to be used as a matrix for hydrogel manufacturing is chitosan (CH), which is a linear polysaccharide obtained by the deacetylation of chitin, naturally occurring in the exoskeletons of crustaceans, insect cuticles and cell walls of some fungi.^4^ Based on its biocompatibility and antibacterial, antifungal, mucoadhesive, and gelling properties, CH is constantly inspiring industrial and academic sectors to develop novel CH-based hydrogels and drug delivery systems.^5,6^ It is well-known that controlled drug delivery systems are a prime stratagem for minimizing both the frequency of therapeutic administration and systematic side effects with high drug content.

In this context, CH hydrogels are known to be very efficient and useful systems for delivering growth factors, cells, and drugs, including anti-inflammatory compounds.^7–11^ Three different mechanisms are described to explain the release of drugs from CH hydrogels: (i) diffusion-controlled, (ii) swelling-controlled, and (iii) chemically controlled release.^12^ The positive effects of drug delivery systems based on CH hydrogels have been demonstrated in many different experimental conditions.^13^ For example, Javdani et al. evaluate the neuroprotective effect of local implantation of a controlled delivery system of CH hydrogel loaded with selenium nanoparticles in rats with spinal cord injury and concluded that this drug delivery system (CH enriched with selenium nanoparticles) seems to play a role in the protection of nerve cells through its anti-inflammatory effect.^14^ Wu et al. found that CH-based composite hydrogel films were able to inhibit inflammatory mediators such as NO, IL-6 and TNF-α, as well as showing antimicrobial activity against Porphyromonas gingivalis, which reinforces their therapeutic potential.^15^ Although many authors have demonstrated the benefits of CH hydrogels enriched with many different anti-inflammatory molecules and drugs, it was not possible to find in the literature any study investigating the effects of the CH hydrogel system for delivering biocompound (with expected anti-inflammatory effects) from marine sponges.

Nowadays, the biocompounds from marine sponges have been widely studied by different authors for their anti-inflammatory properties.^16,17^ Indeed, inflammation is a typical sign associated with different pathological conditions, such as diabetes, atherosclerosis, epilepsy, and neurodegenerative disorders.^18^ Anti-inflammatory compounds from marine sponges have been studied by many researchers both *in vitro* and *in vivo* models.^19^ Around 84 anti-inflammatory substances obtained from marine sponges have been reported. Terpenoids, alkaloids, peptides, and polyketides are some of the major constituents isolated from marine sponges.^17,19^ Mayer et al. demonstrated that five amphilectane metabolites and two semisynthetic derivatives from the marine sponge *Hymeniacidon* sp. (family Halichondriidae) displayed anti-inflammatory properties by inhibiting rat brain microglia thromboxane B2 synthesis via the cyclooxygenase-dependent mechanism.^20^ In addition, the bioactive molecule 9,11-dihydrogracilin A (DHG) isolated from Dendrilla membranosa (family Darwinellidae) showed immunomodulatory and anti-inflammatory effects, reducing cell growth, proliferation, viability, and migration.^21^

Some authors claim that some bioactive compounds from sponges present a significative anti-inflammatory potential, acting on the modulation of various pathways of the inflammatory process, such as inhibiting phospholipase A2 and inhibiting interleukin synthesis.^22,23^ Examples include cavernolide isolated from the species Fasciospongia cavernosa, contignasterol isolated from the species Petrosia contignata and cyclolinteinone from the species Cacospongia linteiformis.^24^ In addition, there are studies showing that steroid-type compounds can also be isolated from sponges, such as clatriol (isolated from the sponge Clathria lissosclera), which has shown anti-inflammatory activity in assays on human blood neutrophil cells and rat mast cells.^25^ Furthermore, Webb et al. demonstrated the anti-inflammatory effects of the compound Perthamide C (a cyclopeptide) isolated from the sponge Theonella swinhoei in *in vitro* studies. Moreover, evidence showed that the sponge genus Dysidea presents a composition abundant in terpenes.^26–29^ Also, according to Williams et al.,^30^ the sponge Dysidea robusta (DR) has the potential to serve as a source of these anti-inflammatory compounds, mainly based on its availability and yield.

In this context, the development of innovative ant-inflammatory drug delivery systems based on CH hydrogels enriched with natural biocompounds of marine sponges is in high demand.^31^ These systems are an alternative to the continued use of anti-inflammatory drugs that can lead to the occurrence of therapy-limiting side effects like gastrointestinal distress, renal toxicity, and even drug resistance.^32^ Then, the hypothesis of the present study is that CH-based hydrogels enriched with biocompounds extracted from DR marine sponges would constitute an appropriate system for delivering anti-inflammatory compounds in *in vitro* studies. The aim of this study was to develop different CH hydrogels (with different compositions of urease and urea) and to characterize the hydrogels. Through the rheological properties of the gels, they were analyzed using time sweep and frequency sweep tests, while their structural properties were examined using Fourrier transform infrared spectroscopy (FTIR) and scanning electron microscopy (SEM). Also, the second aim was to enrich the chosen formulation of the hydrogels with the extracted biocompounds from marine sponges and to evaluate the *in vitro* biological activity of the system using *in vitro* tests viability, cell proliferation, and immunoenzymatic tests to determine the expression of inflammatory cytokines.

## Materials and Methods

### Materials

CH (degree of deacetylation ≥75%, viscosity: ca. 200–400 mPa.s) was purchased from Sigma-Aldrich (St. Louis, MO). Urease (type III from cowpea, purity ≥95%) was obtained from Sigma-Aldrich (St. Louis, MO). Urea solution (purity ≥ 99%) was purchased from Sigma-Aldrich (St. Louis, MO).

### Manufacturing the Hydrogel

For this study, 4 different CH hydrogels were manufactured, with different concentrations of urease and urea (Table 1). To prepare the hydrogels, 2.5% (w/v) low molecular weight CH was dissolved in 1 mL of 0.100 M HCl under magnetic resonance at room temperature. Then, 0.67 mL of urease at a concentration of 7.5 or 10 U/mL (considering 25.920 U/g) dissolved in sterile phosphate-buffered saline (PBS, pH 7.4) was added to the solution. Then, 10 μL of 25 or 50 M urea dissolved in Milli-Q water were added. The solution was kept in agitation for 20 s, as per Tim et al.^33^ Finally, the solution was pipetted (in different volumes according to the test requirements) into molds for gelation.

**Table 1:** Experimental Hydrogel Groups

groups	CH	urease	urea
H1	2.5% w/v	25 U/Ml	7.5 U/mL
H2	2.5% w/v	25 U/mL	10 U/mL
H3	2.5% w/v	50 U/mL	7.5 U/mL
H4	2.5% w/v	50 U/mL	10 U/mL

### Characterization Tests

#### Mass Loss

The mass loss of the hydrogels was assessed using 200 μL of the hydrogel prepared as described above. The samples were then removed from the mold, weighed on a precision scale, and transferred individually to acrylic tubes containing 5 mL of PBS (pH 7.4). The samples were weighed at 1, 5, 10, 15, 30, and 45 days after incubation. The percentage mass loss of the samples was defined as

$$\%\;de\mathrm{grad}ation=\left(P_{f}/P_{i}\right)\times 1000$$

*P*_f_ being the weight of the hydrogel after immersion in PBS, and *P*_i_ the initial weight of the hydrogel samples.^34^

#### Fourier Transform Infrared Spectroscopy

For FTIR analysis, the Jasco spectrometer, model FT/IR-6200, was utilized, with a range between 600–4000 cm^–1^ and 32 scans acquisition.

#### Rheology

Rheological analyses to measure the flow and deformation behavior of materials under controlled conditions were performed using a Physica MCR 101 parallel plate rheometer (Anton Paar, Austria). The tests were conducted at 37 °C. To determine the apparent viscosity of the samples, a controlled cyclic increase in deformation levels was applied, ranging from 0.1 to 50%. This procedure was performed at a constant angular frequency (ω), which varied linearly from 0 to 300 rad/s during the measurement. During the frequency sweeps, the oscillation of the upper plate was maintained at a constant angular frequency (ω), while the deformation amplitude (γ) gradually increased over time.

#### Scanning Electron Microscope

To check the morphological properties of the hydrogels, all the samples were frozen, freeze-dried, refrozen in liquid nitrogen, and fractured to visualize the internal surface. Samples were then mounted on an aluminum base with carbon tape and analyzed with the TM400 electronic mycroscopic instrument (Hitachi, Japan), using a magnification of 300×.

### Marine Sponges and Biocompound Extraction

#### Collecting Marine Sponges

Specimens of the marine sponge Dysidea robusta (Kingdom: Animalia; Phylum: Porifera; Class: Demospongiae; Subclass: Heteroscleromorpha; Order: Poecilosclerida; Family: *Dysideida*e; Genus: *Dysidea*; Species: Dysidea robusta) were collected at Prainha, in Arraial do Cabo, Rio de Janeiro (−22.960072°, −42.018200°) under SISGEN (AEAF480) and SISBio (28.917-1) approval. The sponges were cleaned in seawater and placed in thermal containers filled with seawater. After being transported, the specimens were subjected to three consecutive washes with distilled water to eliminate any remaining cellular residue. After that, they were weighed, cut into tiny pieces, and quickly frozen at −20 °C.

#### Extraction of Biocompounds

The marine sponge was crushed and immersed in ethanol and methanol (EtOH:MeOH 1:1) for 12 h. After filtering, the supernatant was stored for future studies, and the remaining material was washed with MeOH to produce the methanolic extract. A Büchi (Flawil, Switzerland) rotary evaporator, model R-215, containing a vacuum controller model I-300 and a diaphragm pump model V-300 was used to concentrate the extracts. After evaporation, the DRM was resuspended in a hydroalcoholic solution (MeOH:H_2_O—7:3) and subjected to liquid–liquid partitioning in a separation funnel using hexane and ethyl acetate as solvents. After this process, the solvents were evaporated under reduced pressure, generating three partition phases: hexane (DRMH), ethyl acetate (DRMA), and hydroalcoholic (DRMOH). These phases were biomonitored and the DRMH showed the greatest biological activity, which is why it was selected for further fractionation using open column chromatography (2 cm diameter by 2 m height), using Sephadex-LH 20 (GE Healthcare) as the stationary phase and the eluent system proposed by Cardellina^35^ as the mobile phase: 1:4 Hexane:Dichloromethane, 3:2 Dichloromethane:Acetone and 1:4 Dichloromethane:Acetone.^35^ This process yielded 287 samples, which were analyzed by Comparative Thin Layer Chromatography (TLC) silica gel 60 sheets with ALUGRAM Xtra SIL aluminum support, 0.20 mm thick, and UV 254 fluorescence indicator from Macherey-Nagel (Dueren, Germany). The TLC analysis allowed the compounds to be grouped according to the similarity of the spots on the plate, after being revealed under UV light (ultraviolet transilluminator, λ 253 and 365 nm, Spencer) and by ceric sulfate reagent (Ce_2_(SO_4_)_3_). In this way, it was possible to obtain 10 groups (DRMH1–DRMH10), which were again biomonitored, resulting in the choice of DRMH4 due to its greater biological activity. DRMH4 was fractionated in an open column (4 cm in diameter by 30 cm high), using silica gel 60 (Merck) as the stationary phase and a gradient of dichloromethane:methanol—100% DCM, 99:1, 98:2, 95:5, 90:10, 80:20, 70:30, 60:40, 50:50, 100% MeOH (LabSynth, Diadema, SP, Brasil) as the mobile phase. At the end of this process, 171 samples were obtained and then analyzed by TLC and grouped into seven subgroups (DR1–DR7) which were analyzed in this study (Figure 1).

**Figure 1:**
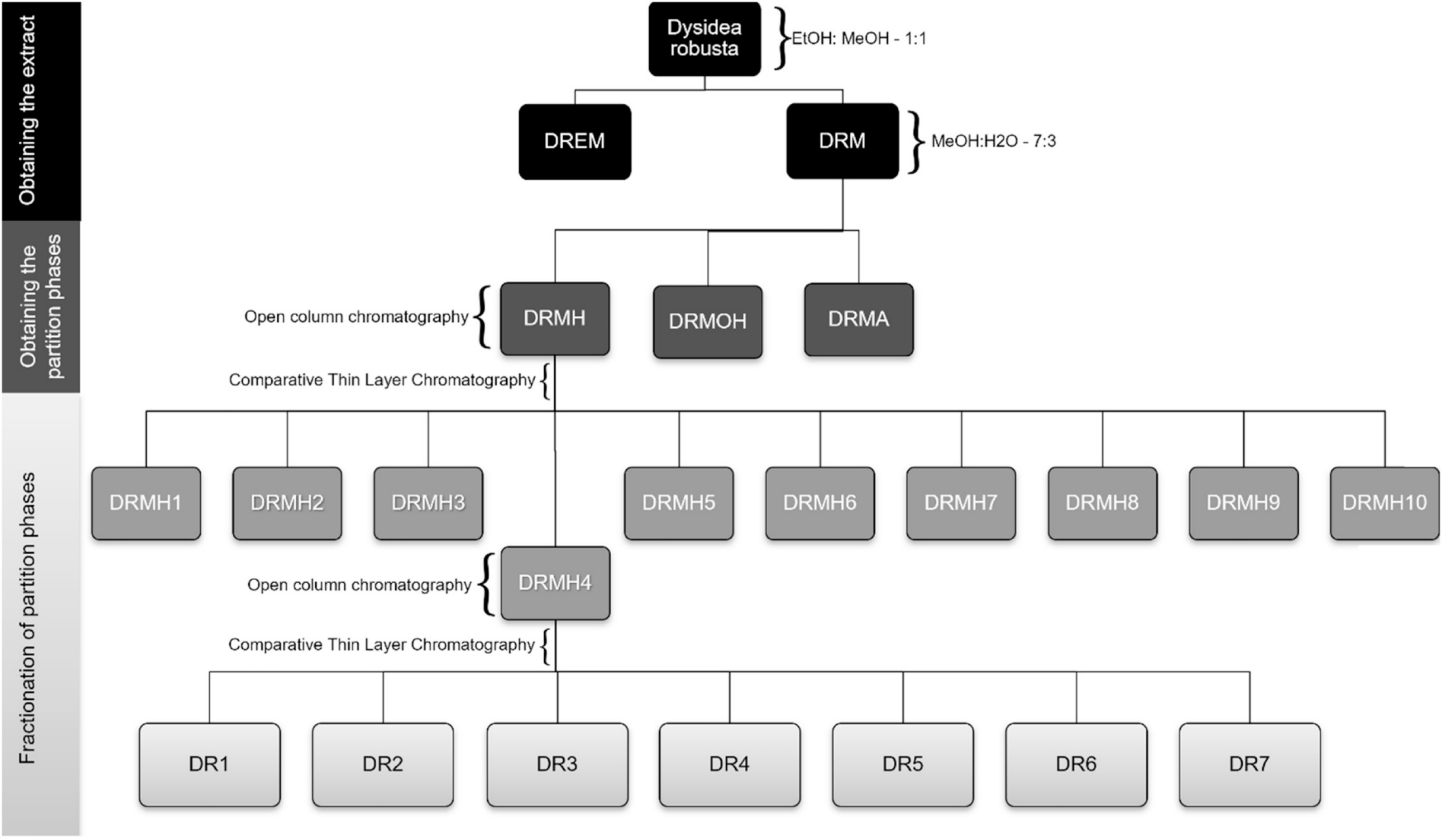
Flowchart of the extraction and fractionation process of bioactive compounds from the marine sponge Dysidea robusta.

#### Biocompound Incorporation into the Hydrogel

Samples of the selected hydrogel were then enriched with each of the 7 compounds (DR1–DR7) from the last extraction process of biocompounds extracted from the DR at a concentration of 0.250 mg/mL (Table 2). After mixing the CH solution with the urease, the compound was incorporated, and after homogenization, the urea solution was added and kept stirring for 20 s and placed in the molds.

**Table 2:** Biocompound Incorporation into the Hydrogel

groups	hydrogel composition	biocompound (0.250 mg/mL)
H3DR1	H3	DR1
H3DR2	H3	DR2
H3DR3	H3	DR3
H3DR4	H3	DR4
H3DR5	H3	DR5
H3DR6	H3	DR6
H3DR7	H3	DR7

#### FTIR and SEM with the hydrogels enriched with biocompounds

To check whether the addition of bioactive compounds to the hydrogel influenced its physical and chemical characteristics, FTIR and SEM analyses were carried out, as described above.

#### Drug release test

50 mL of PBS at 37.5 °C were added to each sample of the chosen hydrogel (H3) enriched with each of the 7 DR compounds for the drug release tests. Aliquots of 500 μL were collected at regular intervals, ranging from 15 min to 7 days. Since the partition phases are complex mixtures of different bioactive compounds, quantification was performed using the peak area of the dominant compound in each different DR phase, as identified by high-performance liquid chromatography (HPLC). For the DR1, DR2, DR3, DR4, DR5, DR6, and DR7 fractions, the peak areas corresponding to the retention times of 1.797, 1.825, 2.313, 2.741, 2.730, 3.829, and 2.917 min, respectively, were analyzed. The HPLC assays were carried out with an Agilent 1260 Infinity II liquid chromatograph equipped with a Poroshell 120 EC-C18 column (4.6 × 100 mm^2^, 4 μm). Detection was performed using an Agilent diode array detector. The mobile phase consisted of an 80% (v/v) acetonitrile solution in water with a flow rate of 1 mL/min. The effluent was monitored at a wavelength of 210 nm, and the injection volume was set at 10 μL. A calibration curve for the bioactive compounds was prepared within the concentration range of 0.152–12 μL, diluted in 2 mL of PBS.

#### Nonlinear regression analysis (Korsmeyer–Peppas model)

The drug transport constants (*k*) and diffusion exponents (*n*) of the different groups were determined by fitting the drug release test data to the Korsmeyer–Peppas equation (https://www.ptfarm.pl/pub/File/Acta_Poloniae/2010/3/217.pdf).

$$M_{t}/M_{\infty }=kt^{n}$$

*M_t_* being the amount of drug released in time (*t*), *M*_∞_ is the total amount of drug incorporated into the hydrogel, and *k* is the release constant, which depends on the characteristics of the polymer and the drug. Microsoft Office Excel (Microsoft Corporation, Redmond) was used to determine *K* and *n*. In addition, the range of *M*_*t*_/*M*_∞_ was 0–60%.^36^

### *In Vitro* Assays

#### Cell culture

Murine fibroblasts (L929), macrophages (RAW.264.7) (BCRJ, RJ, Brazil), and chondrocytes derived from the articular cartilage of Wistar rats from the cell bank of the tissue engineering laboratory at Unifesp (Unifesp, São Paulo, Brazil). The fibroblasts and chondrocytes were cultured in DMEM (Dulbecco’s modified Eagle’s medium) (Vitrocell, Embriolife, Campinas, SP, Brazil) with 2 mM glutamine, 100 U/mL penicillin, 100 mg/mL streptomycin, and supplemented with 10% fetal bovine serum (FBS) (Vitrocell, Embriolife, Campinas, SP, Brazil). The macrophages were grown in RPMI culture medium (Vitrocell, Embriolife, Campinas, SP, Brazil) with 2 mM glutamine, 100 U/mL penicillin, 100 mg/mL streptomycin, and supplemented with 10% fetal bovine serum (FBS) (Vitrocell, Embriolife, Campinas, SP, Brazil). All cells were cultured under standard conditions (37 °C in a humid atmosphere containing 5% CO_2_). The experimental groups consisted of the control group (CG), H3 group, and H3 groups enriched with each compound from the DR bioactive compounds (H3DR1 to H3DR7).

#### Preparation of material for indirect contact tests

All the hydrogels from the groups described above (H3, H3DR1–H3DR7) were manufactured in the laminar flow cabinet, and then a 5% concentration of hydrogel from each group (w/v) was integrated into Falcon tubes containing DMEM or RPMI culture medium (according to the cell line to be used in each analysis) supplemented with 10% fetal bovine serum. These preparations were then incubated in a CO_2_ incubator set at 37 °C. After a 24 h incubation period, membrane filters with 0.22 μm pores from Kasvi (Brazil) were used to filter the extract to ensure purity and remove any particulate material. The cells were exposed to this extract for *in vitro* analyses.

#### Metabolic activity

In accordance with ISO 10993-5 (2009),^37^ biocompatibility was assessed using the cell viability test by indirect contact, exposing L929 cells and chondrocytes to 500 μL of the extract prepared as described above. For this analysis, a cell concentration of 1 × 10^4^ was seeded in a 48-well plate. After the experimental periods of 1, 3, and 6 days, 500 μL of a 10% alamarBlue solution was added to each well and incubated in the dark for 4 h. Next, 200 μL of the solution (in triplicate) were aliquoted into 96-well plates to be analyzed by reading the absorbance using a microplate spectrophotometer (Bio-Tek Instruments, 570–600 nm). From the values obtained, cell viability was calculated as a percentage of alamarBlue, according to the manufacturer’s instructions.

#### Cell proliferation assay

DNA quantification was carried out on the same plates seeded with L929 and chondrocytes for the viability analysis. After two freezing and thawing cycles (−80 and 25 °C), 200 μL of a freshly prepared working solution was added to each well containing 10 μL of sample or standard DNA. The plate was kept in the dark for 5 min. The fluorescent signal was read using a microplate spectrophotometer (GloMax Discover Microplate Reader, Promega. 504–531 nm).

#### Enzyme-linked immunosorbent assay

To evaluate the anti-inflammatory potential of the hydrogel enriched with the DR1–DR7 compounds, cultures of RAW 265.7 macrophage cells treated with 2 μg/mL LPS for 24 h were used to induce the M1 phenotype. The cells were cultured and exposed indirectly to the extract. 1 × 10^3^ cells were seeded in each well of 96-well plates. At the end of the experimental periods of 1 and 3 days, the RPMI medium was collected. The samples were dosed with the cytokines TNF-α and IL-6. High-affinity microplates were sensitized with anticytokine monoclonal antibodies and left overnight at room temperature. After blocking with PBS, the plates were washed, the supernatants were added, and standard curves of recombinant cytokines were made. The plates were kept at room temperature for two h and then washed again. Biotinylated anticytokine antibodies were added and kept for another hour at room temperature. Cytokines were measured using an ELISA immunoassay, following the manufacturer’s recommendations. The results were expressed as optical density.

#### Chemical characterization of D5

Nuclear Magnetic Resonance spectra ^1^H and ^13^C (operating at 500 and 125 MHz, respectively) were recorded on a Varian INOVA 500 spectrometer (Palo Alto, CA) using CDCl_3_ (Sigma-Aldrich, St. Louis, MO) as solvent and TMS as internal standard. The obtained spectra were processed in the MestReNova software, version 14, provided by the company Mestrelab Research S. L. (Santiago de Compostela, GAL, Spain). High-resolution positive mode mass spectra were recorded by the Ultra Performance Liquid Chromatograph Prep LC 4000 (UV–vis detector) with Mass Spectrometer Acquity UPLC/Q-Tof micro, Waters-Micromass (Mississauga, ON, Canada).

## Results

### Characterization of CH hydrogels

#### Mass loss

Figure 2 shows the changes in the mass of the CH hydrogels in the different periods evaluated. On day 1, the mass of the hydrogels fell by 22.2 ± 1.92% for H1, 21.1 ± 1.89% for H2, 22.8 ± 2.31% for H3, and 20.6 ± 2.04% for H4, with no statistical differences observed between the groups. On the fifth day, the values were 16.1 ± 1.72% for H1, 15.6 ± 1.47% for H2, 16.2 ± 0.67% for H3, and 16.7 ± 1.86% for H4. On day 10, there were reductions of 33.5 ± 2.31% for H1, 30.5 ± 0.88% for H2, 23 ± 2.86% for H3, 24.6 ± 1.18% for H4 in relation to the original mass, with significant differences between groups H1 vs H3 and H4, and H2 vs H3 and H4. On day 15, the values were 40.6 ± 3.90% for H1, 41.0 ± 3.64% for H2, 36.6 ± 1.20% for H3 and 36.0 ± 2.03% for H4. After 30 days, there was a reduction in mass of 67.6 ± 1.11% for H1, 67.3 ± 2.11% for H2, 52.6 ± 3.58% for H3 and 51.4 ± 4.77% for H4, with significant differences between H1 vs H3 and H4 and H2 vs H4. Finally, on day 45, the decrease in mass was 72.7 ± 4.11% for H1, 72.4 ± 3.42% for H2, 64.8 ± 1.95% for H3, and 63.9 ± 1.61% for H4, with significant differences between H1 vs H3 and H4 and H2 vs H3 and H4.

**Figure 2:**
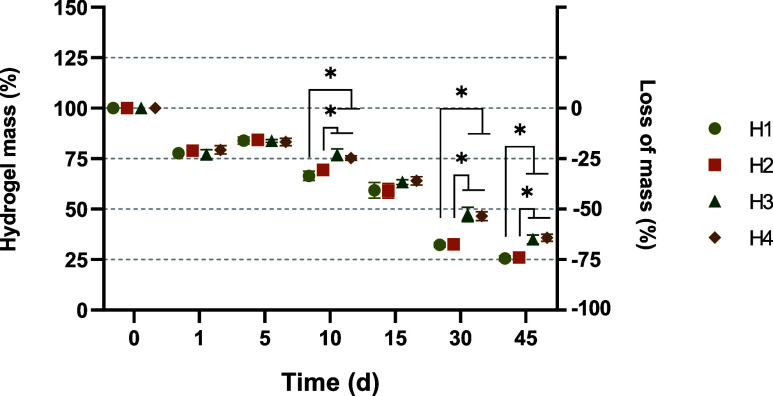
Percentage of decrease of the mass of the CH hydrogels at different times (results expressed as mean ± SD for 3 repetitions). ANOVA and Tukey’s posthoc: Statistical differences represented by *P < 0.005.

#### FTIR

Figure 3 shows the FTIR spectra of the CH hydrogel samples. For the samples, the band observed at 3253 cm^–1^ is attributed to overlapping O–H and N–H stretching (υ) vibrations, while the band at 2876 cm^–1^ corresponds to aliphatic C–H stretching.^38^ These bands are affected by the urea/urease concentrations used to form the hydrogels, which are more prominent in the H3 sample. The characteristic amide I band, associated with C=O stretching, appears at 1623 cm^–1^. The peak at 1513 cm^–1^ indicates N–H bending (δ) vibrations, specifically related to the N-acetylation of CH.^39^ The band at 1380 cm^–1^ is linked to C–H bending vibrations, reflecting the polymer backbone curvature. The band located at 1307 cm^–1^ corresponds to the C–N stretching of amide III.^40^ The antisymmetric stretching of the C–O–C bridge is assigned to the peak at 1150 cm^–1^, while the bands at 1068 cm^–1^ and 1017 cm^–1^ correspond to skeletal vibrations, particularly C–O stretching within the CH structure.^41^ The band at 1068 cm^–1^ represents more structured or organized regions of the CH (C–O–C), whereas the band at 1017 cm^–1^ reflects vibrations in more flexible or less organized areas of the polymer (C–O–H).^42^ Both bands involve C–O stretching, but in slightly different structural contexts. For the H1, H2, and H3 samples, the band at 1017 cm^–1^ shows slightly higher intensity than the 1068 cm^–1^ band, while in the H4 sample, this pattern is reversed.

**Figure 3:**
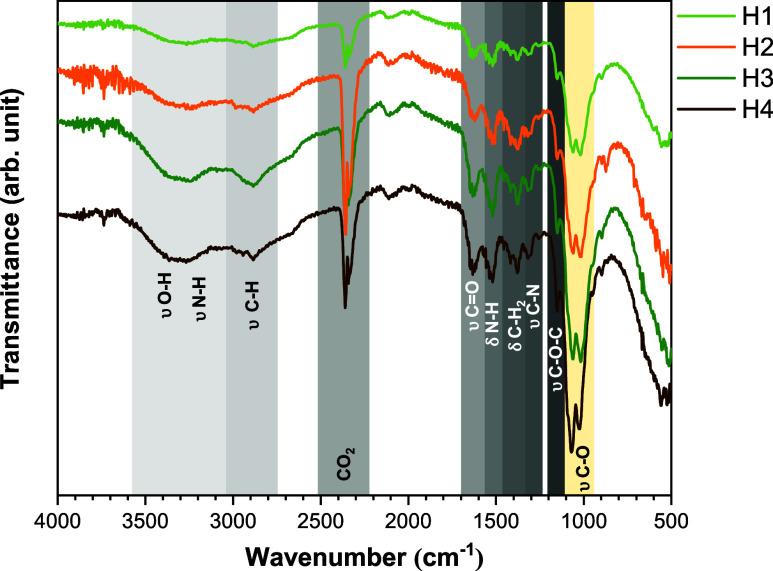
FTIR spectra of CH hydrogels.

#### Rheology

The gelation time was analyzed by using rheology through time sweep tests (Figure 4A). It was observed that the ratio of urea to urease affects the gelation time, which was determined by extrapolating the rising curve with the baseline of each sample. For the H1, H2, H3, and H4 samples, the gelation times were 12.3, 21.6, 33.7, and 44.0 min, respectively. Thus, the higher the urease concentration relative to urea, the shorter the gelation time. These results align with the pH variation, as the transition from acidic to neutral pH occurs at approximately the same times. Figure 4B shows the rheological analysis of a frequency sweep for the different hydrogel groups, with the parameters *G*′ (elastic modulus) and *G*″ (viscous modulus) being analyzed as a function of frequency. In the group analyses, the *G*′ is significantly higher than the *G*″ across the entire frequency range. The *G*′ curve remains nearly constant, indicating a predominantly elastic behavior. *G*″ is also relatively stable but with much lower values, indicating low viscosity. It can also be observed that lower urease concentrations (H1 and H2) produce gels with higher *G*′ values than those with higher concentrations. These results suggest that the CH hydrogels exhibit more elastic characteristics, making them suitable for applications in which the hydrogel is expected to retain its shape under varying stress conditions.

**Figure 4:**
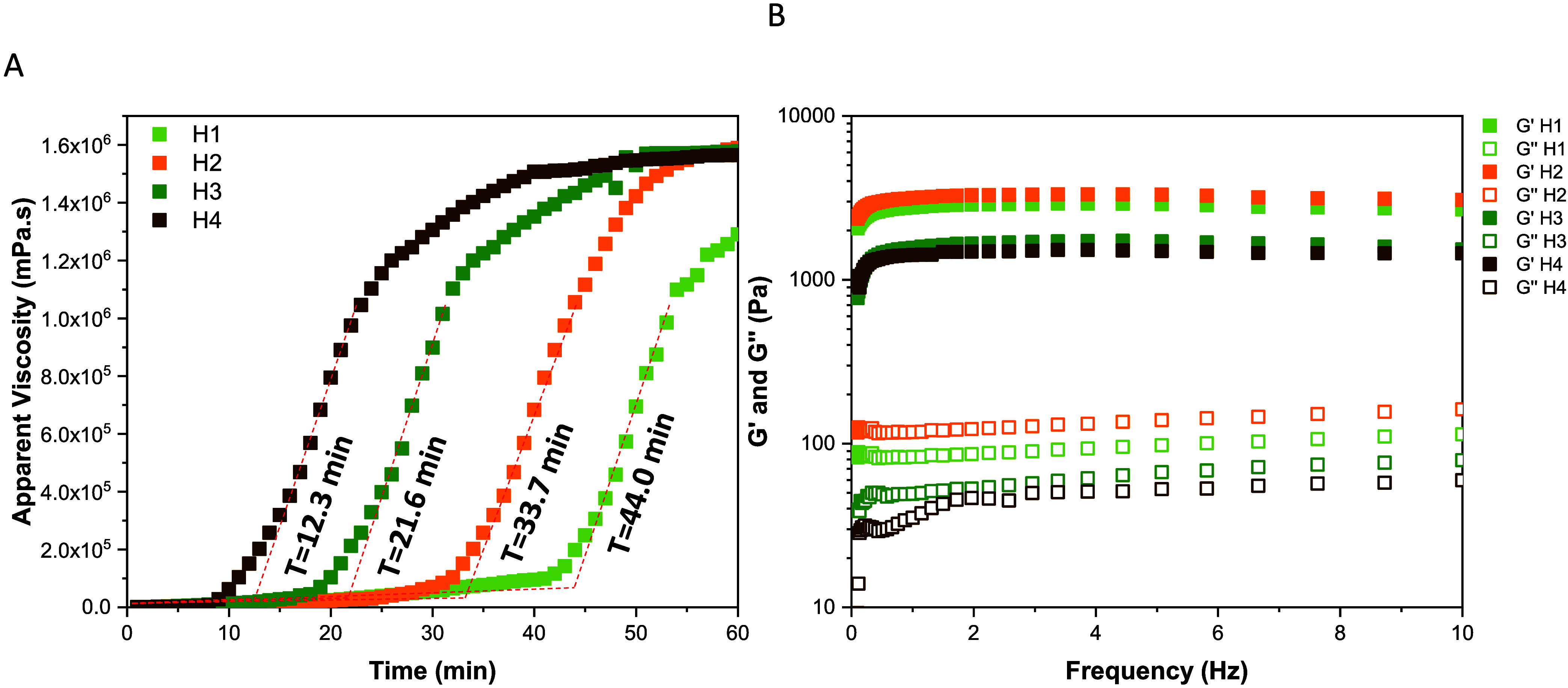
Dynamic changes in viscoelastic properties of CH hydrogels by rheology using (A) time sweep and (B) frequency sweep tests.

#### SEM

SEM was used to analyze the morphologies of the hydrogels (Figure 5). At the magnification of 300×, it is possible to identify a porous and lamellar structure in the groups H1 and H2, with H1 presenting a rougher appearance, while the lamellar network in H2 is less evident, presenting a greater surface smoothness. H3 presented a more compact surface, with a notable reduction in the lamellar characteristics while H4 has a more homogeneous and dense structure, with less evidence of porosity or visible fibers.

**Figure 5:**
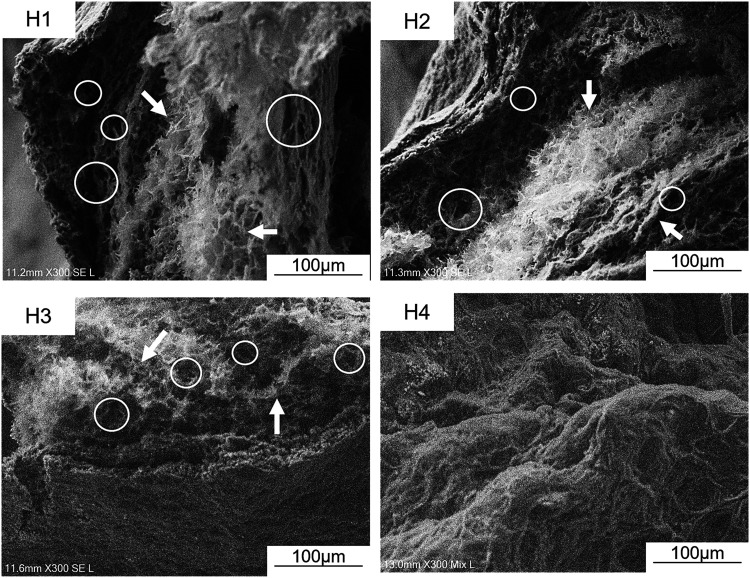
Freeze-dried microstructures of CH hydrogels observed using SEM. The asterisks indicate the presence of pores, and the arrows indicate lamellae.

#### FTIR and SEM of CH hydrogels with DR biocompounds

The H3 hydrogel was selected for the incorporation of enriched bioactive compounds from the sponge. The choice of hydrogel composition was based on the characterization results, focusing on the group of hydrogels that demonstrated sufficient gelation time for potential injections and minimal mass loss.

##### FTIR

Figure 6 shows the FTIR spectra for the H3 hydrogel after the addition of various compounds extracted from DR (DR1–DR7). However, when the C–O stretching bands are examined in detail, slight intensity variations can be noted. The 1068 cm^–1^ band is more intense in the hydrogels containing compounds DR2, DR5, and DR7, while for compounds DR1, DR3, and DR7, the band at 1017 cm^–1^ appears slightly more intense. For compound DR4, no differences are observed compared to those of the H3 hydrogel.

**Figure 6:**
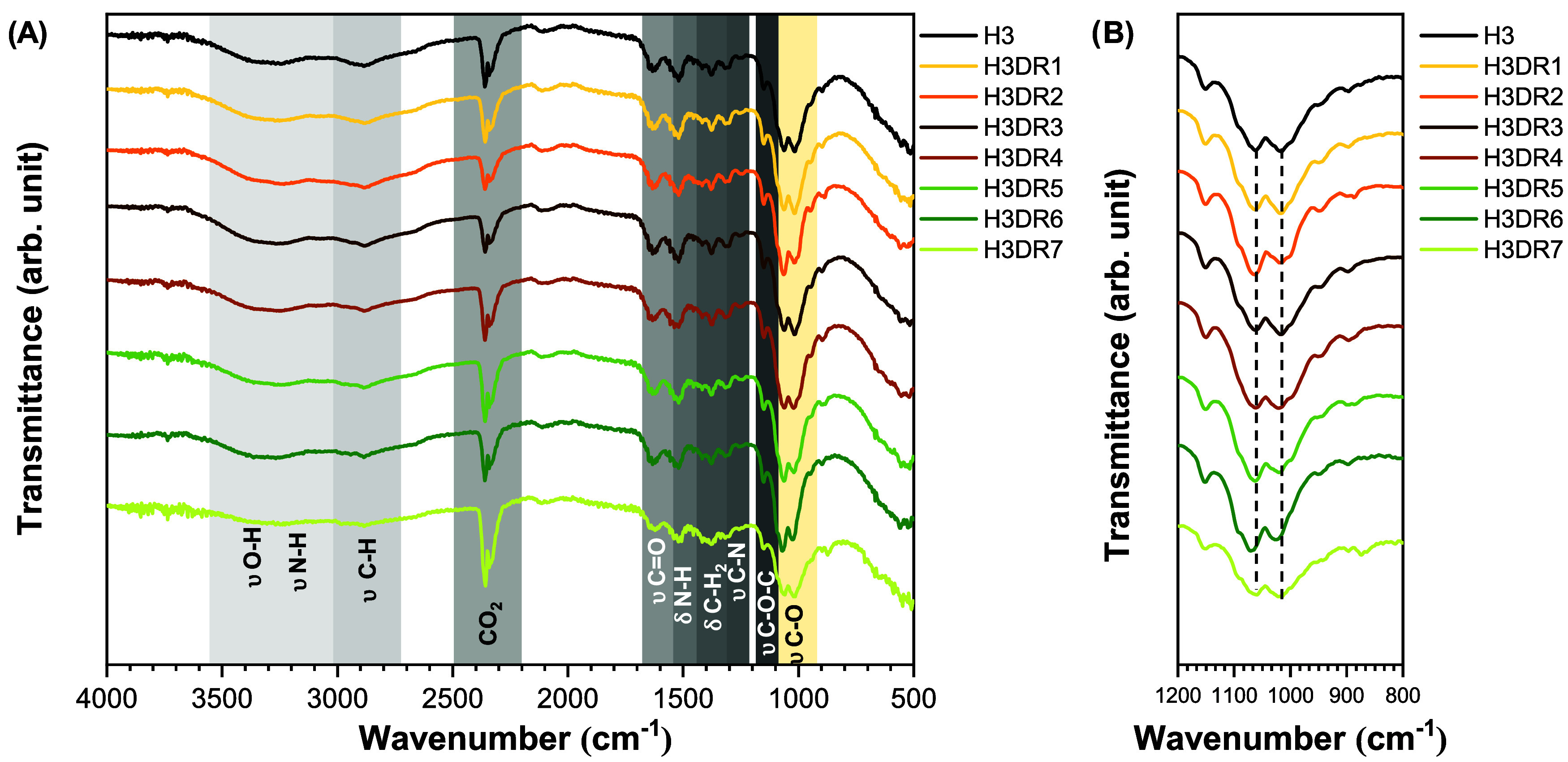
FTIR spectra of H3 hydrogels loaded with bioactive compounds from DR1–DR7. (A) Scanning spectrum from 4000–500 cm^–1^. (B) Enlargement of the spectrum from 1200–800 cm^–1^ with the C–O stretching bands.

##### SEM

Figure 7 shows the images of the microstructures of H3 after loading of DR bioactive compounds (DR1–DR7) observed using SEM (including H3 used for comparison). The introduction of DR1 (H3DR1) results in a more compact and dense structure with no apparent lamellae aspect compared to H3. Furthermore, H3DR2 presents a fibrous structure and H3DR3 presents a more spongy and irregular appearance, with visible areas of collapse and contraction. H3DR4, H3DR5, and H3DR6, on the other hand, exhibit a highly dispersed matrix, with thinner, more widely spaced lamellar structures and high porosity. Furthermore, H3DR7 appears to have a more compact structure, with fibrillar and lamellar areas coexisting.

**Figure 7:**
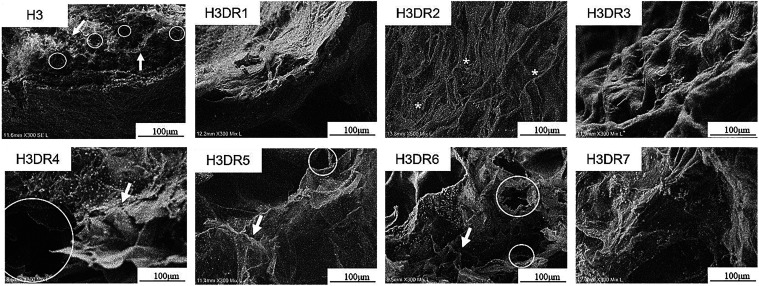
Freeze-dried microstructures of H3 hydrogels loaded with bioactive compounds from DR1–DR7 observed using SEM. Circles indicate the presence of pores, arrows indicate lamellae, and asterisks indicate fibers.

### Drug release test

Figure 8 shows the release profile of the experimental groups over 7 days, analyzed by HPLC. Each compound was evaluated based on its release time from the H3 hydrogel. For compounds DR1, DR2, and DR6, approximately 74% of the bioactive compound was released in the first 8 h, with an additional 8–16% release over the next 7 days (86% for DR1, 82% for DR2, and 90% for DR6). The DR4 compound had 85% of its release in the first 8 h and 94% in the 7-day period. For compounds DR3, DR5, and DR7, the release at the 8 h mark was 50, 44, and 54%, respectively. However, a steady gradual release was observed over the following days, reaching 89, 74, and 82% for DR3, DR5, and DR7, respectively.

**Figure 8:**
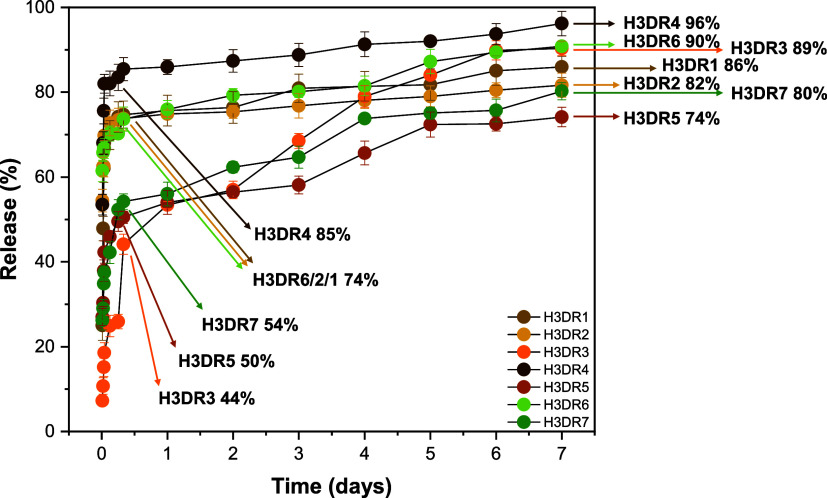
Bioactive release profile over time for H3 hydrogels loaded with bioactive compounds extracted from DR (DR1–DR7).

### Nonlinear data fitting using the Korsmeyer–Peppas model

From the data fit, the transport constants and transport exponents were determined (see Figure S1) using the Korsmeyer–Peppas model, which is an empirical model widely used to describe drug release from polymeric matrix systems. The data showed that DR1, DR3, DR5, and DR7 had the highest diffusion exponents, being 0,17, 0,37, 0,12 and 0,15 respectively. While DR2, DR4 and DR6 had lower values, 0,6, 0,7, and 0,4 respectively (Table 3).

**Table 3:** Biocompound Incorporation into the Hydrogel

groups	drug transport constants (*k*)	diffusion exponents (*n*)
H3DR1	1.43	0.17
H3DR2	1.28	0.06
H3DR3	0.77	0.37
H3DR4	1.49	0.07
H3DR5	0.89	0.12
H3DR6	1.24	0.04
H3DR7	0.94	0.15

### *In vitro* analysis

#### Metabolic activity

Figure 9 provides data on the cell viability of chondrocytes (Figure 9A) and fibroblasts (Figure 9B) exposed to the hydrogel and bioactive compounds for 1, 3, and 6 days. It is important to highlight that none of the tested fractions exhibited toxicity toward fibroblasts, as shown in Figure S2. The cell viability of chondrocytes on day 1 resulted in rates of 100.00 ± 0.00% for CG, 98.92 ± 7.65% for H3, 99.35 ± 2.97% for H3DR1, 94.90 ± 2, 66% for H3DR2, 98.59 ± 2.44% for H3DR3, 97.95 ± 11.87% for H3DR4, 100.47 ± 12.63% for H3DR5, 93.34 ± 7.33% for H3DR6 and 92.86 ± 7.39% for H3DR7. On day 3, the values were 100.00 ± 0.00% for the CG, 93.88 ± 9.59% for H3, 97.99 ± 5.91% for H3DR1, 94.99 ± 9.65% for H3DR2, 102.86 ± 4.02% for H3DR3, 87.49 ± 4.08% for H3DR4, 92.30 ± 2.69% for H3DR5, 93.21 ± 6.03% for H3DR6 and 90.17 ± 5.66% for H3DR7. For the last period (day 6), the results were 100 ± 0.00% for the CG, 97 ± 8.63% for H3, 98 ± 13.49% for H3DR1, 99 ± 4.40% for H3DR2, 101 ± 5.01% for H3DR3, 91 ± 5.34% for H3DR4, 106 ± 7.44% for H3DR5, 102 ± 7.18% for H3DR6 and 106 ± 17.63% for H3DR7. No statistical difference was found when comparing the groups studied in relation to the CG for any experimental period (Figure 9A).

**Figure 9:**
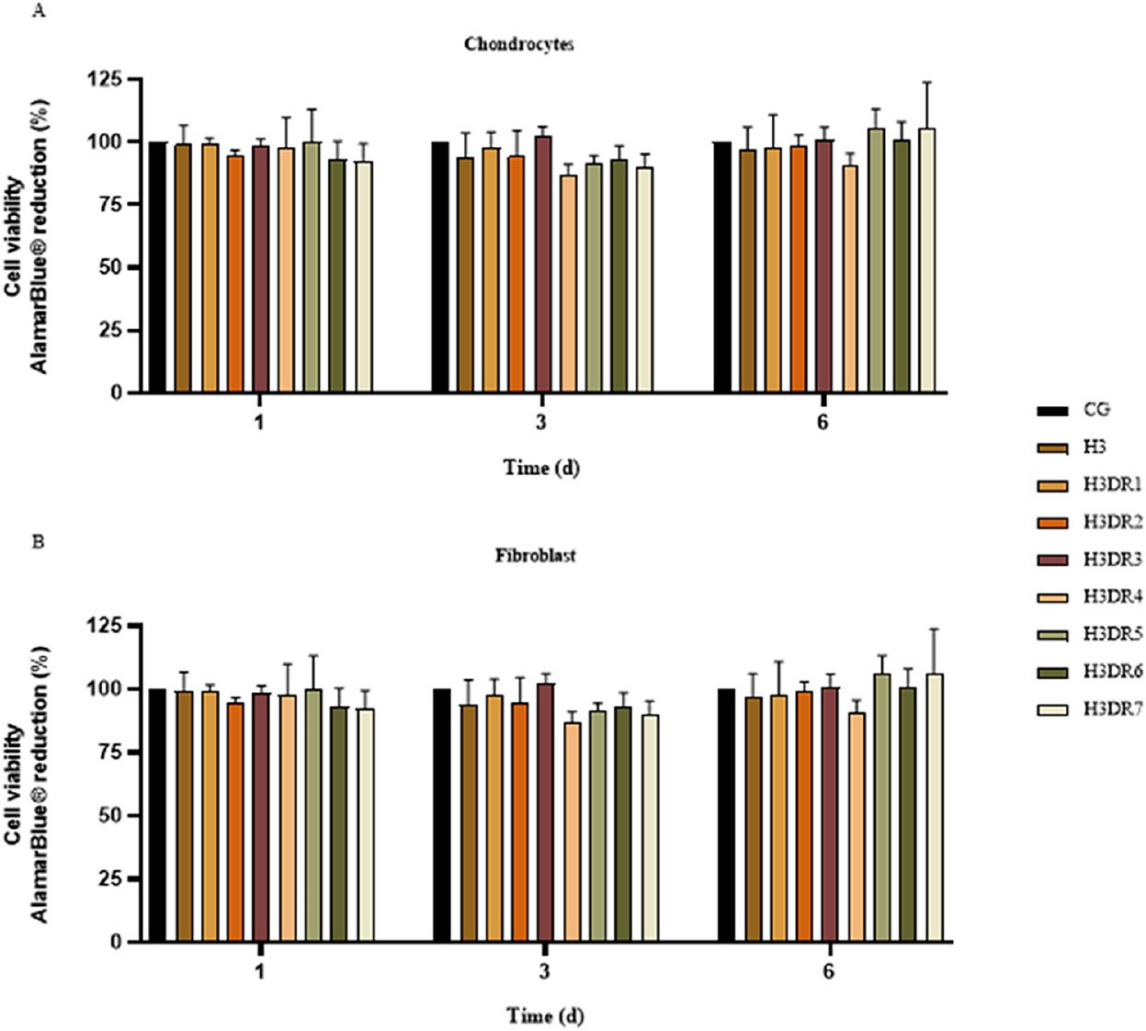
Metabolic activity of (A) chondrocytes and (B) fibroblasts exposed to bioactive compounds derived from marine sponges DR over time. ANOVA and Tukey’s posthoc: Statistical differences represented by *P < 0.005.

For the analysis carried out on the fibroblast cells, on day 1, cell viability showed the following values: 100.00 ± 0 for CG, 96.42 ± 7.32% for H3, 97.50 ± 4.40% for H3DR1, 94.76 ± 2.74% for H3DR2, 97.16 ± 4.92% for H3DR3, 96.88 ± 10.96% for H3DR4, 104.31 ± 16.70% for H3DR5, 93.18 ± 7.46% for H3DR6 and 92.69 ± 7.52% for H3DR7. On day 3, the results were 100.00 ± 0.00% for CG, 91.27 ± 9.22% for H3, 95.38 ± 5.82% for H3DR1, 92.38 ± 9.39% for H3DR2, 100.25 ± 4.28% for H3DR3, 84.88 ± 4.39% for H3DR4, 89.69 ± 2.62% for H3DR5, 90.60 ± 6.53% for H3DR6 and 87.56 ± 5.70% for H3DR7. Finally, on day 6, fibroblasts showed viability of 100.00 ± 0.00% for the CG, 94.48 ± 8.82% for H3, 97.86 ± 13.87% for H3DR1, 99.25 ± 4, 54% for H3DR2, 101.33 ± 5.17% for H3DR3, 90.46 ± 5.52% for H3DR4, 106.60 ± 7.74% for H3DR5, 101.78 ± 7.41% for H3DR6 and 106.45 ± 18.20% for H3DR7. As with the chondrocytes, no statistical differences were found when compared to the CG (Figure 9B).

#### Proliferation

Figure 10 shows the results of the proliferation of chondrocytes (Figure 10A) and fibroblasts (Figure 10B) for all of the experimental groups over different periods. Analysis of the chondrocytes on day 1 revealed that the amount of dsDNA was 557.7 ± 42.5 ng/mL for the CG, 531.7 ± 37.8 ng/mL for H3, 652.3 ± 12.7 ng/mL for H3DR1, 509.7 ± 17.5 ng/mL for H3DR2, 531.7 ± 37.8 ng/mL for H3DR3, 563.3 ± 61.4 ng/mL for H3DR4, 479.7 ± 44.7 ng/mL for H3DR5, 428.7 ± 33.6 ng/mL for H3DR6 and 355.7 ± 72.8 ng/mL for H3DR7, with no statistical differences between the groups studied compared to the control. On day 3, the values were 953.3 ± 55.1 ng/mL for the CG, 720.3 ± 27.1 ng/mL for H3, 723.7 ± 30.7 ng/mL for H3DR1, 737.3 ± 124.7 ng/mL for H3DR2, 720.3 ± 27.1 ng/mL for H3DR3, 664.0 ± 106.7 ng/mL for H3DR4, 938.3 ± 70.8 ng/mL for H3DR5, 1143.3 ± 124.2 ng/mL for H3DR6 and 644.7 ± 110.3 ng/mL for H3DR7, with significant differences being found between CG vs H3DR1 and H3DR3. Finally, on day 6, the results were 1156.7 ± 60.3 ng/mL for CG, 841.7 ± 103.6 ng/mL for H3, 1056.7 ± 50.3 ng/mL for H3DR1, 580.3 ± 22.4 ng/mL for H3DR2, 1106.7 ± 100.2 ng/mL for H3DR3, 789.0 ± 36.0 ng/mL for H3DR4, 783.3 ± 78.4 ng/mL for H3DR5, 1017.7 ± 85.1 ng/mL for H3DR6 and 956.0 ± 120.1 ng/mL for H3DR7, with the following statistical differences: CG vs H3DR2, H3DR5, and H3DR6.

**Figure 10:**
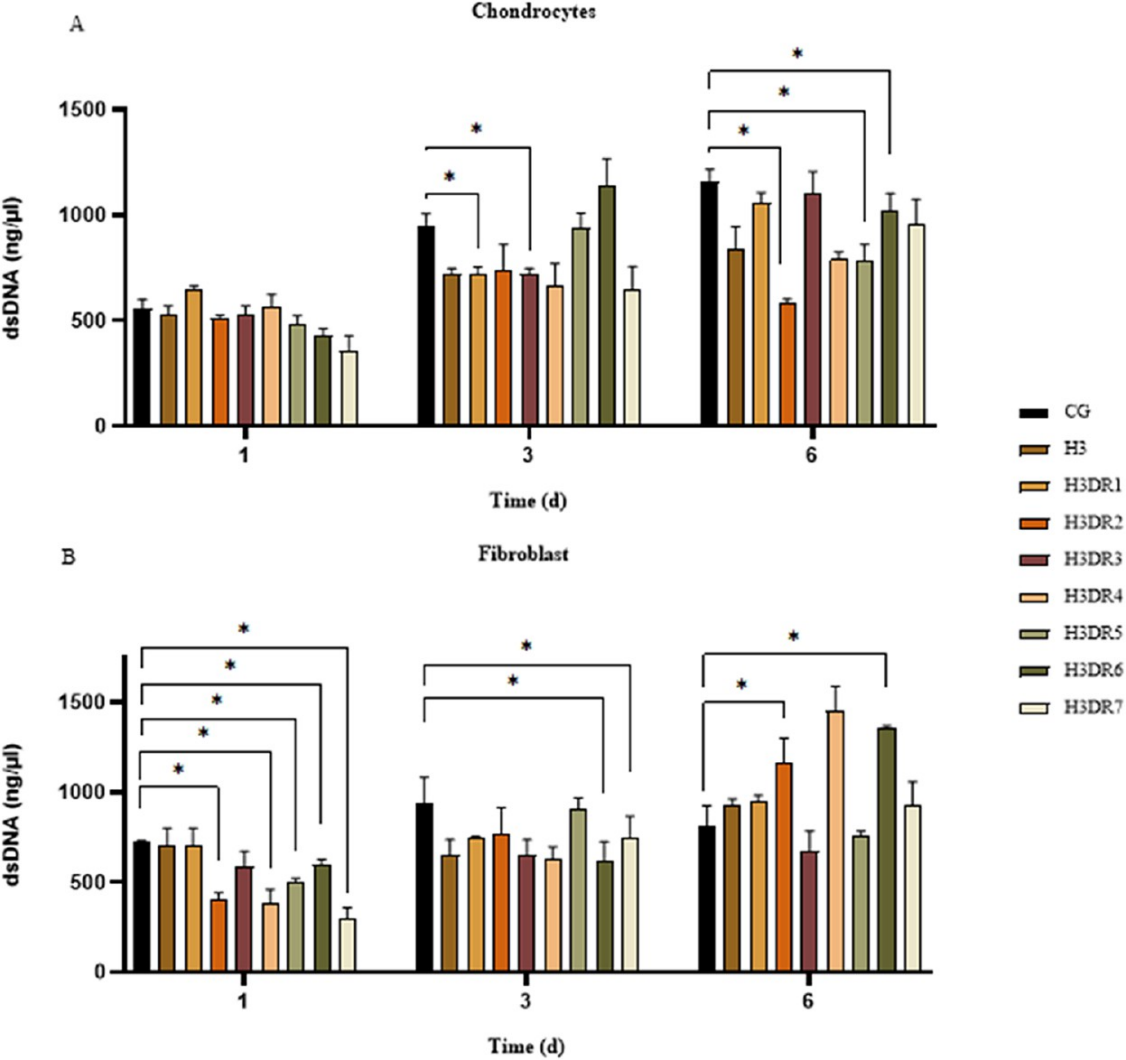
Proliferation of (A) chondrocytes and (B) fibroblasts exposed to bioactive compounds derived from marine sponges DR over time. ANOVA and Tukey’s posthoc: Statistical differences represented by *P < 0.005.

As for fibroblast proliferation, on day 1, the results were 727.3 ± 3.1 ng/mL for CG, 699.7 ± 99.5 ng/mL for H3, 699.7 ± 99.5 ng/mL for H3DR1, 408.3 ± 34.9 ng/mL for H3DR2, 583.7 ± 88.3 ng/mL for H3DR3, 386.3 ± 75.4 ng/mL for H3DR4, 496.0 ± 26.2 ng/mL for H3DR5, 596.3 ± 28.7 ng/mL for H3DR6 and 296.0 ± 63.5 ng/mL for H3DR7, with statistical differences being found when comparing the CG vs H3DR2, H3DR4, H3DR5, H3DR6 and H3DR7. In the second period, day 3, the values were 940.3 ± 143.8 ng/mL for CG, 653.0 ± 84.8 ng/mL for H3, 744.3 ± 10.2 ng/mL for H3DR1, 768.3 ± 146.4 ng/mL for H3DR2, 653.0 ± 84.8 ng/mL for H3DR3, 626.7 ± 70.2 ng/mL for H3DR4, 910.7 ± 57.0 ng/mL for H3DR5, 612.7 ± 110.8 ng/mL for H3DR6 and 742.3 ± 125.0 ng/mL for H3DR7 with statistical differences between CG vs H3DR5 and H3DR6. Finally, day 6 showed the following results: 815.0 ± 110.1 ng/mL for the CG, 932.3 ± 31.0 ng/mL for H3, 947.0 ± 36.8 ng/mL for H3DR1, 1161.3 ± 139.2 ng/mL for H3DR2, 670.3 ± 113.9 ng/mL for H3DR3, 1451.7 ± 137.0 ng/mL for H3DR4, 760.0 ± 22.0 ng/mL for H3DR5, 1353.3 ± 15.3 ng/mL for H3DR6 and 927.3 ± 132.6 ng/mL for H3DR7, with statistical differences found between CG vs H3DR2 and H3DR6.

#### Enzyme-linked immunosorbent assay

Figure 11 shows the expression levels of IL-6 (Figure 11A) and TNF-α (Figure 11B) in macrophages for all groups at both experimental periods. It is possible to observe that CG (1049.33 ± 11.05 pg/mL) showed the highest levels of IL-6 expression on day 1, revealing statistical differences when compared to all of the other groups. Also, the values found for IL-6 expression for the other groups were: 338.18 ± 7.53 pg/mL for H3, 313.81 ± 7.69 pg/mL for H3DR1, 342.39 ± 18.67 pg/mL for H3DR2, 362.38 ± 8.79 pg/mL for H3DR3, 311.15 ± 7.90 pg/mL for H3DR4, 113.25 ± 9.12 pg/mL for H3DR5, 227.26 ± 5.78 pg/mL for H3DR6, and 338.98 ± 8.29 pg/mL for H3DR7. The following statistically significant differences were found among the experimental groups: H3 vs H3DR1, H3DR3, H3DR4, H3DR5, and H3DR6; H3DR1 vs H3DR2, H3DR3, H3DR5, H3DR6, and H3DR7; H3DR2 vs H3DR3, H3DR4, H3DR5, and H3DR6; H3DR3 and H3DR4, H3DR5, H3DR6, and H3DR7; H3DR4 vs H3DR5, H3DR6 and H3DR7; H3DR5 vs H3DR6 and H3DR7 and H3DR6 vs H3DR7.

**Figure 11:**
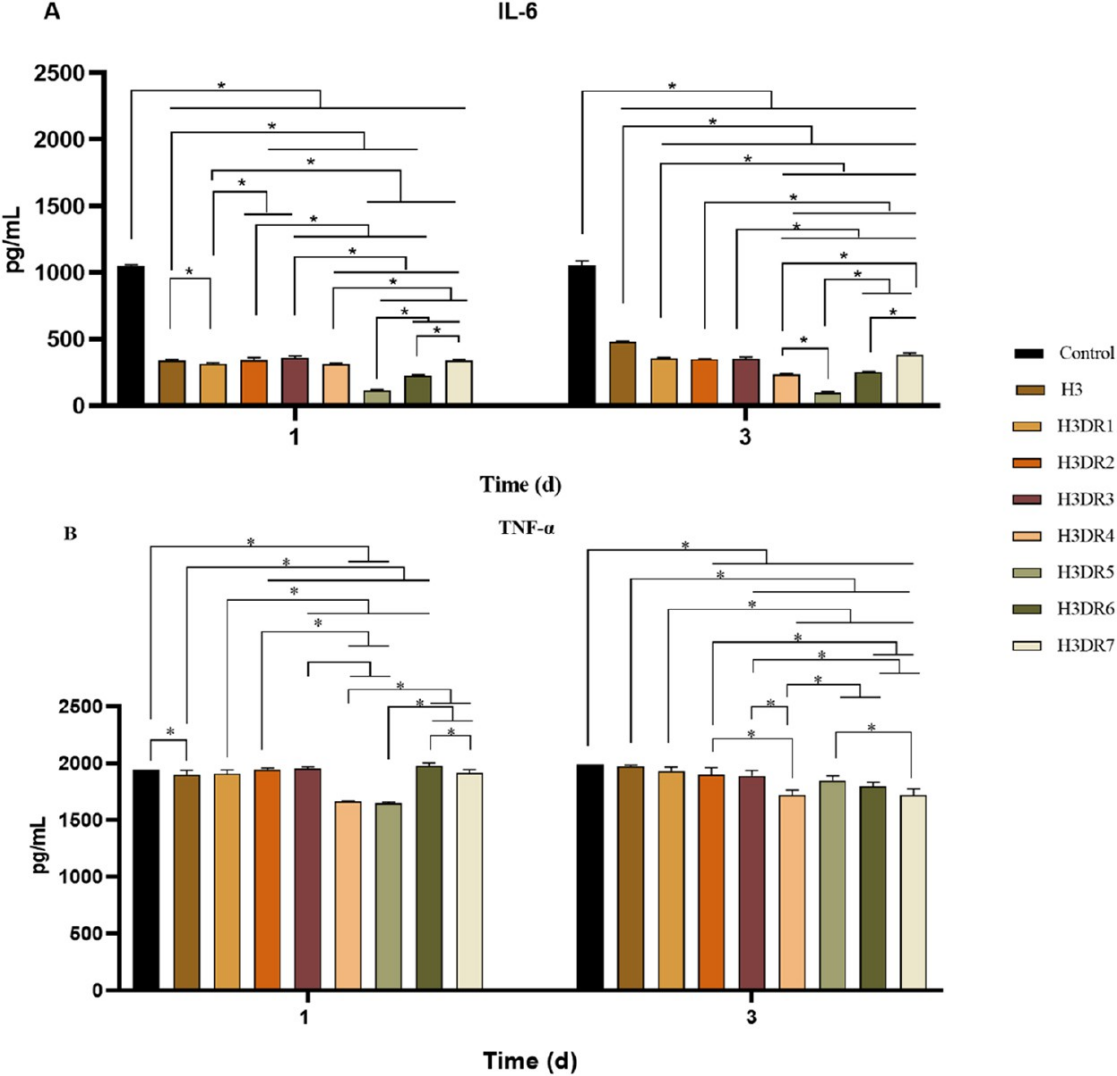
ELISA analysis of cytokine levels in macrophage cultures across different experimental groups. The graphs depict (A) IL-6 and (B) TNF-α. ANOVA and Tukey’s posthoc: Statistical differences represented by *P < 0.005.

On day 3, for CG an expression level of 1057 ± 30.79 pg/mL was found. Also, for the experimental groups, the following values were observed: for H3 480.11 ± 6.65 pg/mL, for H3DR1 354.53 ± 7.86 pg/mL, for H3DR2 348.88 ± 4.25 pg/mL, for H3DR3 353.85 ± 11, 53 pg/mL, for H3DR4 236.38 ± 6.80 pg/mL, for H3DR5 98.56 ± 6.34 pg/mL, for H3DR6 252.16 ± 6 pg/mL and for H3DR7 384.1 ± 13.71 pg/mL. A statistically significant difference was found among CG and all of the other experimental groups. Moreover, there were also statistical differences among the following treatment groups: H3 vs all the others; H3DR1 vs H3DR4, H3DR5, H3DR6 and H3DR7; H3DR2 vs H3DR4 to H3DR7; H3DR3 vs H3DR4 to H3DR7; H3DR4 vs H3DR5 and H3DR7; H3DR5 vs H3DR6 and H3DR7 and H3DR6 and H3DR7.

As for TNF-α expression on day 1, the values found were: 1945 ± 0 pg/mL for the CG, 1898.33 ± 40.15 pg/mL for H3, 1908.33 ± 33.82 pg/mL for H3DR1, 1942.16 ± 16.97 pg/mL for H3DR2, 1953.66 ± 13, 88 pg/mL for H3DR3, 1665.33 ± 5.12 pg/mL for H3DR4, 1649 ± 10.58 pg/mL for H3DR5, 1979 ± 23.96 pg/mL for H3DR6 and 1915.66 ± 29.50 pg/mL for H3DR7. In addition, statistically significant differences were CG vs H3, H3DR4, and H3DR5; H3 vs H3DR2 and H3DR6; H3DR1 vs H3DR3 and H3DR6; H3DR2 vs H3DR4 and H3DR5. H3DR3 vs H3DR4 and H3DR5; H3DR4 vs H3DR6 and H3DR7; H3DR5 vs H3DR6 and H3DR7 and H3DR6 vs H3DR7.

On the third day, TNF-α level values were: CG 1991 ± 0 pg/mL, H3 1972.66 ± 11.05 pg/mL, H3DR1 1929.91 ± 35.25 pg/mL, H3DR2 1901, 66 ± 59.38 pg/mL, H3DR3 1889 ± 48 pg/mL, H3DR4 1718.83 ± 43.81 pg/mL, H3DR5 1846.83 ± 40.91 pg/mL, H3DR6 1796.66 ± 37.77 pg/mL H3DR7 expressing 1720.5 ± 54.05 pg/mL. The following statistical differences were observed: CG vs H3DR2 and H3DR7, H3 vs H3DR7, H3DR1 vs H3DR4 and H3DR7, H3DR2 vs H3DR4, H3DR6 and H3DR7, H3DR3 vs H3DR4, H3DR6 and H3DR7, H3DR4 vs H3DR5 and H3DR6, and finally, H3DR5 vs H3DR7.

#### Rheology of Hydrogel H3DR5

Rheological analysis was performed on the hydrogel incorporating the DR5 fraction, which contained the extract with the most prominent anti-inflammatory activity. This analysis was conducted because interactions between the components of the DR5 fraction and chitosan (CH) could significantly influence the hydrogel’s viscoelastic behavior and gelation time. The results are presented in Figure 12. The gelation time of the H3DR5 sample exhibited a slight increase compared to the pure H3 hydrogel, rising from 21.6 to 22.2 min. Additionally, the *G*′ and *G*″ values showed a modest increase, indicating slightly enhanced mechanical properties.

**Figure 12:**
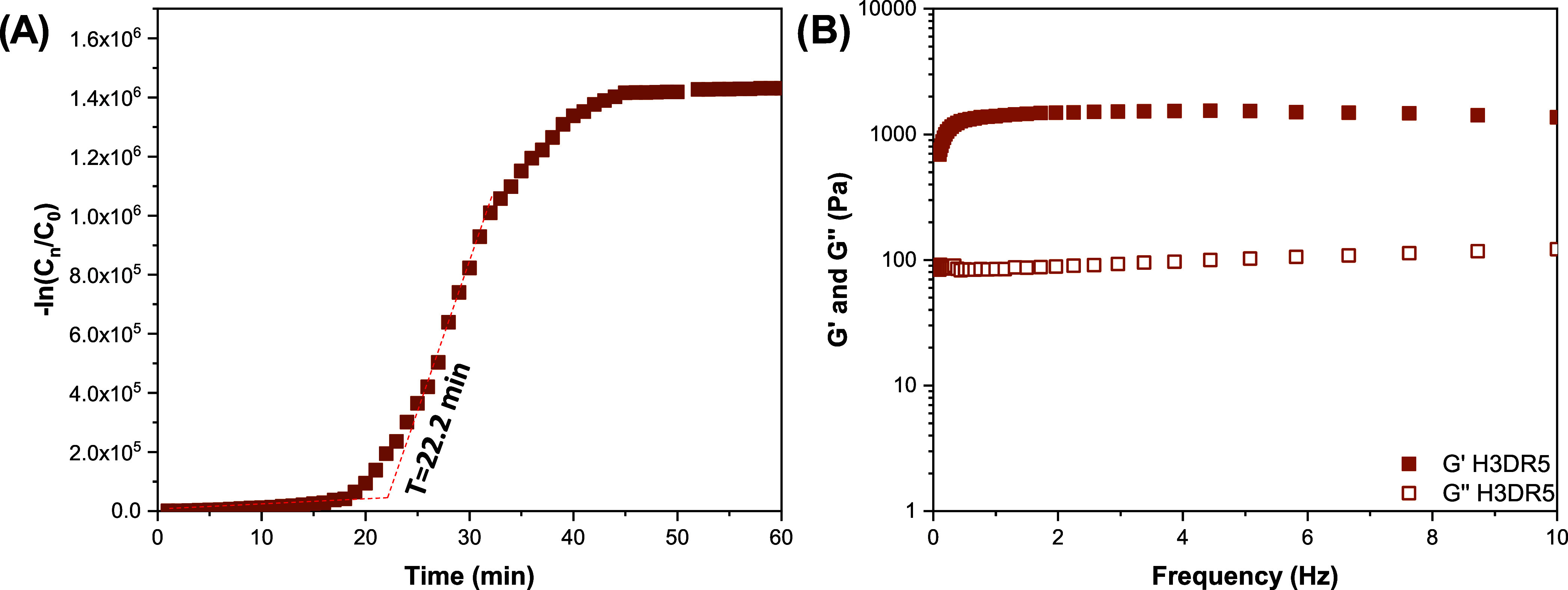
Dynamic changes in viscoelastic properties of H3DR5 by rheology using (A) time sweep and (B) frequency sweep tests.

#### Chemical characterization of D5

As the hydrogel incorporated with fraction D5 displayed higher anti-inflammatory activity in comparison to other fractions, the chemical characterization of the main compound was tentatively performed. ^13^C and DEPT NMR spectra showed a carbonyl carbon at δ 173.9 and several sp^2^ methine carbons ranging from δ 130.6 and 128.1, characteristic of unsaturated side chains. These data associated with the presence of two carbinolic carbons at δ 68.3 (CH_2_) and 65.0 (CH) suggested the occurrence of 1,3-diacyl glycerol derivative.^43^ This proposal was confirmed by analysis of ^1^H NMR spectrum due to the presence of signals at δ 5.36 (m) attributed to hydrogens of olefinic carbons, at δ 4.15 (m) assigned to glyceryl moiety, one intense singlet at δ 1.25 (s).^44^ Finally, analysis of ESI-HRMS data with displayed [M + Na]^+^ peak at *m*/*z* 917.5709 compatible with molecular formula of C_59_H_106_O_5_, allowed the identification of the main compound from fraction D5 as depicted in Figure 13.

**Figure 13:**
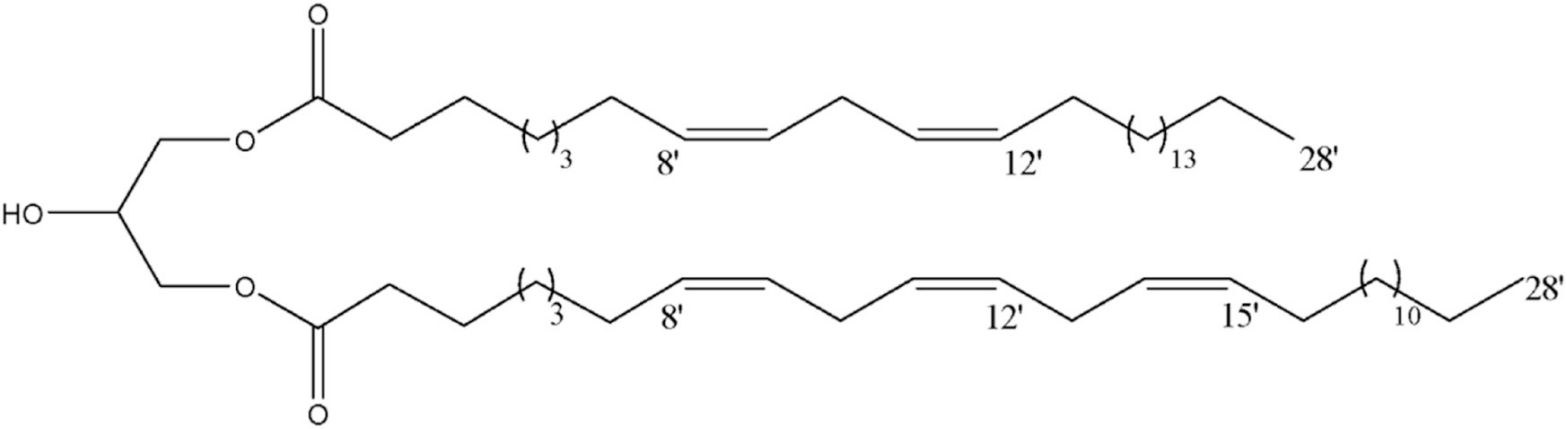
2-Hydroxy-3-(((8Z,11Z)-octacosa-8,11-dienoyl)­oxy)­propyl (8Z,11Z,14Z)-octacosa-8,11,14-trienoate identified in the most active fraction of Dysidea robusta.

## Discussion

This study evaluated the morphological and physical properties of different CH hydrogel formulations to determine the most suitable system for delivering biocompounds from marine sponges. Characterization tests revealed a gradual loss of mass in CH hydrogels over time, more pronounced in H1 and H2, while FTIR and SEM analyses confirmed the porous structure of the hydrogels. Rheology tests demonstrated that gelation time and elasticity varied with the urease/urea ratio with the longest gelation time and highest elasticity being observed for H1. *In vitro assays* revealed different patterns in the release of the compounds, in which DR1, DR2, and DR6 showed rapid initial release, followed by slow release. DR4 had a similar behavior but with a higher initial release. In contrast, DR3, DR5, and DR7 exhibited more sustained release from the beginning. Cell viability and proliferation tests confirmed the biocompatibility of all of the formulations. Furthermore, enzyme immunoassays revealed that H3DR5 significantly reduced IL-6 levels compared with other groups, with TNF-α expression notably lower in this group, particularly on day 1.

Mass stability results revealed a consistent mass loss of all formulations throughout the experimental periods with H1 and H2 showing the most pronounced reduction (approximately 73%). This phenomenon is likely attributed to the enzymatic cleavage of CH molecules, which not only releases CH fragments, resulting in hydrogel weight loss, but also decreases the cross-link density. This reduction in density may allow for greater water absorption, potentially increasing the hydrogel’s wet weight.^45^ At the end of the experiment, H3 and H4 retained approximately 36% of their initial mass, highlighting their potential as sustained drug delivery vehicles due to their slower degradation profiles. This result contrasts with the findings of Yan et al. who observed that CH hydrogels with higher concentrations of urease they studied showed a higher degradation rate, while the lower concentrations resulted in greater structural stability.^46^ The degradation rate of hydrogels is a crucial factor in drug delivery systems, as it allows for the prolonged and controlled release of drugs into tissues, thus reducing the need for frequent injectable interventions.^47^

As degradation rates are influenced by the amounts of urea and urease used, different structural properties are expected, depending on the applied concentration. These structural changes are evident in FTIR analysis, where H3 and H4 showed greater C–H stretching, and C–O changes were more pronounced in H4.^40,42,48^ In SEM analyses, H1, H2, and H3 showed a mesoporous structure suitable for bioactive release. In contrast, H4 presented a dense and less porous structure, being less suitable for this application, reinforcing the choice of H3 as the ideal carrier.^49,50^

The variation in the concentrations of cross-linking agents also influences the time of transformation from the liquid phase to the gel, assessed by rheology, which analyzes the flow and deformation of the gels.^51^ It was observed in this analysis that the urease/urea ratio and the CH concentration influence the gelation rate.^33,52^ Urea catalyzes the conversion of urea into ammonia and carbon dioxide, increasing the pH, while urea as a substrate determines the amount of ammonia released.^53^ Higher concentrations of urease or urea accelerate the increase in pH, promoting faster gelation.^54^ Considering the initial gel manipulation process, sample H3, which presented a gelation time of approximately 20 min, was selected for its potential for injectable application while still in the liquid phase for subsequent gel formation. Regarding the deformation analyses, lower urease concentrations (H1 and H2) produced gels with higher G’, due to slower gelation, allowing a more structured network. Higher concentrations led to less elastic and softer gels^52,53,55^

After the analysis of the data of the characterization tests, the H3 formulation was the most suitable for constituting the drug delivery system with marine biocompounds. First, hydrogel H3 exhibited an optimal gelation time, lower mass loss, and a more porous structure compared to the other groups. These characteristics are vital, as they maintain the integrity of the hydrogel during its application and the drug release process.

Modifications in the gel formulation, such as the incorporation of biocompounds, can significantly alter its microstructure and, consequently, its properties, as demonstrated by FTIR and SEM analyses, which revealed changes in the microstructure of the H3 hydrogels after the incorporation of DR biocompounds. It is likely that the presence of several classes of biocompounds, such as fatty acids, terpenes or flavonoids, directly influenced their interaction with the hydrogel polymers.^56,57^ More hydrophilic compounds may have promoted greater porosity, as observed in H3DR4, H3DR5, and H3DR6, while more hydrophobic ones may have resulted in denser structures, as seen in H3DR1 and H3DR7.^58–60^

Analysis of the release profiles of the DR compounds from the H3 hydrogel indicated that the release of the bioactives follows a Fickian diffusion mechanism, as described by the Korsmeyer–Peppas model.^36^ The *n* exponent obtained for each compound varied between 0.04 and 0.37, which characterizes a diffusive transport controlled mainly by passive diffusion through the porous structure of the hydrogel without significant influence from polymer relaxation or matrix degradation. This behavior suggests that the bioactive compounds diffuse through the hydrogel following a concentration gradient, with minimal structural changes in the matrix over time.^61,62^ This pattern, known as burst release, is characterized by a rapid initial release, which can be attributed to the high water content and large pore sizes in these materials.^63,64^ Furthermore, this release profile may have promising clinical implications, as the initial rapid release may increase bioactive penetration, while the sustained release ensures a prolonged therapeutic effect^49,50^

*In vitro* analyses showed that all samples maintained cell viability and proliferation above 84%, meeting the criteria of ISO 10993–5:2009, which requires at least 70% viability compared to CG.^37^ The literature highlights biological properties of CH, such as biocompatibility, biodegradability, and absence of immunogenicity, which make it ideal for biomedical applications, including drug delivery^4,9,65,66^ The hydrogels enriched with biocomposites also showed no cytotoxic effects. Previous studies corroborate these results, such as Tommonaro et al. who evaluated sesquiterpenoids delivered by hydrogels using *in vitro* studies and demonstrated nontoxic results.^67^

Regarding the anti-inflammatory potential of the compounds studied, it is known that inflammatory cytokines are fundamental in immune responses and healing.^68^ Among them, IL-6 is essential in acute inflammation, facilitating the release of other cytokines and promoting the transition to a repairing environment.^69,70^ However, high levels of IL-6 can impair healing and increase the risk of infection.^69^ In this study, IL-6 expression was significantly reduced in all the treated groups compared to the CG on days 1 and 3, showing the inhibitory effect of the treatments applied on this pro-inflammatory cytokine. Corroborating these findings, a previous study demonstrated that cyanogramide compounds, isolated from Actinoalloteichus—a sponge-derived actinomycete—were able to inhibit the release of IL-6 in LPS-stimulated RAW264.7 cells by inhibiting the JAK-STAT pathway.^71^ Despite this, the differences observed in the reduction of IL-6 between the groups can be attributed to the unique chemical characteristics of each biocomposite. Interestingly, H3DR5 showed a higher reduction in IL-6 which may suggest that this biocompound was more effective in interfering with inflammatory pathways, such as inhibiting macrophage activation playing a crucial role in this process^72^ The chemical variation between the fractions reinforces the importance of studying each group in isolation in order to identify which specific compounds or combinations have the greatest therapeutic potential.

Another important cytokine in the process of regulating inflammatory responses is TNF-α. This cytokine is involved in key processes, such as the recruitment and activation of cells in tissues. In addition, TNF-α can stimulate the release of other pro-inflammatory cytokines, amplifying the inflammatory cascade, which makes it a prime target for therapeutic intervention.^73^ It is important to note that groups H3, H3DR4, and H3DR5 in the first period and most groups, except H3 and H3DR1, in the second period showed differences when compared to the CG. However, TNF-α levels remained high throughout the experimental period after exposure to the biocompounds, which could be attributed to the fact that this cytokine is associated with a more resistant and multifactorial inflammatory response, involving different regulatory mechanisms However, the levels of TNF-α were kept high during the experimental time after exposure to biocompounds which may be attributed to the fact that this cytokine is associated with a more resistant and multifactorial inflammatory response, involving different regulatory mechanisms.^74,75^ The study by Chaudhari et al. reinforces the anti-inflammatory profile of biocompounds extracted from sponges, showing that fractions from Xestospongia carbonaria, Sarcotragus fetidus and Spongia obscura significantly reduced IL-6 levels in RAW 62 macrophages, while the effects on TNF-α were less significant.^76^

In view of the above, the H3 hydrogel and the H3DR5 bioactive group were considered to be the most promising formulations in this study. The H3 hydrogel demonstrated ideal characteristics for drug delivery systems, including adequate gelation time, lower mass loss, and porous structure, which favor the stability and sustained release of bioactive compounds, and the interaction between the CH and DR5 partition phase does not alter the rheological behavior of the sample. Additionally, the H3DR5 group stood out for its significant anti-inflammatory effects, showing a higher reduction in IL-6 levels while maintaining excellent biocompatibility. As D5 fraction was the most active in this model, the chemical characterization was performed by ^1^H and ^13^C NMR, and ESI-HRMS, allowing the identification of 2-hydroxy-3-(((8Z,11Z)-octacosa-8,11-dienoyl)­oxy)­propyl (8Z,11Z,14Z)-octacosa-8,11,14-trienoate, a Very Long Chain Polyunsaturated Fatty Acid (VLCPUFAs), that can be present in up to ten percent of marine sponges.^77^ The incorporation of long-chain fatty acids in cell membranes can change the fluidity and influence early signal transduction in macrophages and T cells. Furthermore, they also can be responsible for the inhibitory effect of inflammation, decreasing IL-6 expression,^78^ which corroborates the results obtained in our experiments. These findings align with the growing interest in utilizing hydrogels as platforms for the controlled release of natural anti-inflammatory agents. Consequently, the combined potential of marine-derived compounds and hydrogel-based delivery systems presents an attractive avenue for further research and development in tissue engineering and regenerative medicine.

## Conclusions

In this study, CH hydrogels were synthesized with urea/urease and enriched with bioactive compounds extracted from the marine sponge Dysidea robusta, aiming to evaluate the efficacy of the hydrogel as a delivery system and the anti-inflammatory potential of the compounds. The H3 stood out for its stable physical and morphological properties essential for the controlled release of bioactive compounds. *In vitro* assays confirmed the biocompatibility and anti-inflammatory action of the compounds, with emphasis on the reduction of IL-6 by the H3DR5 group, which has 2-hydroxy-3-(((8Z,11Z)-octacosa-8,11-dienoyl)­oxy)­propyl (8Z,11Z,14Z)-octacosa-8,11,14-trienoate as its major component. This group also preserved the ideal viscoelastic properties of the hydrogel. Despite the promising results, additional *in vivo* studies are needed to validate the long-term efficacy and clinical applicability.

## Supplementary Material



## Notes

Supporting Information containing graphs of nonlinear data fitting using the Korsmeyer–Peppas model and metabolic activity of fibroblasts exposed to bioactive compounds derived from DR marine sponges. (PDF)
