# Supplementary material for: Chitosan Hydrogels Enriched with Biocompounds Extracted from Marine Sponges: Potential to Modulate the Inflammatory Process in an In Vitro Study
Source: ACS Omega. 2025 Jun 12;10(24):25605–20. doi: 10.1021/acsomega.5c01171 (PMC12199092; doi:10.1021/acsomega.5c01171)
Supplement: Supplementary file 1 [file ao5c01171_si_001.pdf]

Chitosan hydrogels enriched with biocompounds extracted from marine sponges: potential to modulate the inflammatory process in an *in vitro* study

Mirian Bonifacio[1]; Cíntia C.S. Martignago[1]; Dalete C.S. Souza[2]; Homero Garcia-Motta[1]; Laís C. Souza-Silva[1]; Beatriz Soares-Silva[1] Karolyne S.J. Sousa[1]; Anabella .P. Rosso[2]; João H.G. Lago[2]; Alessandra M. Ribeiro[1]; Marcelo Assis[1]; Renata N. Granito[1,]; Ana C. M. Rennó[1,*]

[1] Department of Biosciences, Federal University of São Paulo (UNIFESP), Santos, SP, 11015020, Brazil.
[2] Center for Natural and Human Sciences, Federal University of ABC (UFABC), Santo André - SP, 09280-560, Brazil.

***Corresponding author:**
Ana Rennó, Federal University of São Paulo - Unifesp, Department of Biosciences, Rua Silva Jardim 136, Vila Matias, Santos -SP, Brazil
E-mail: a.renno@unifesp.br

SUPPORTING INFORMATION

Non-linear data fitting using the Korsmeyer-Peppas model

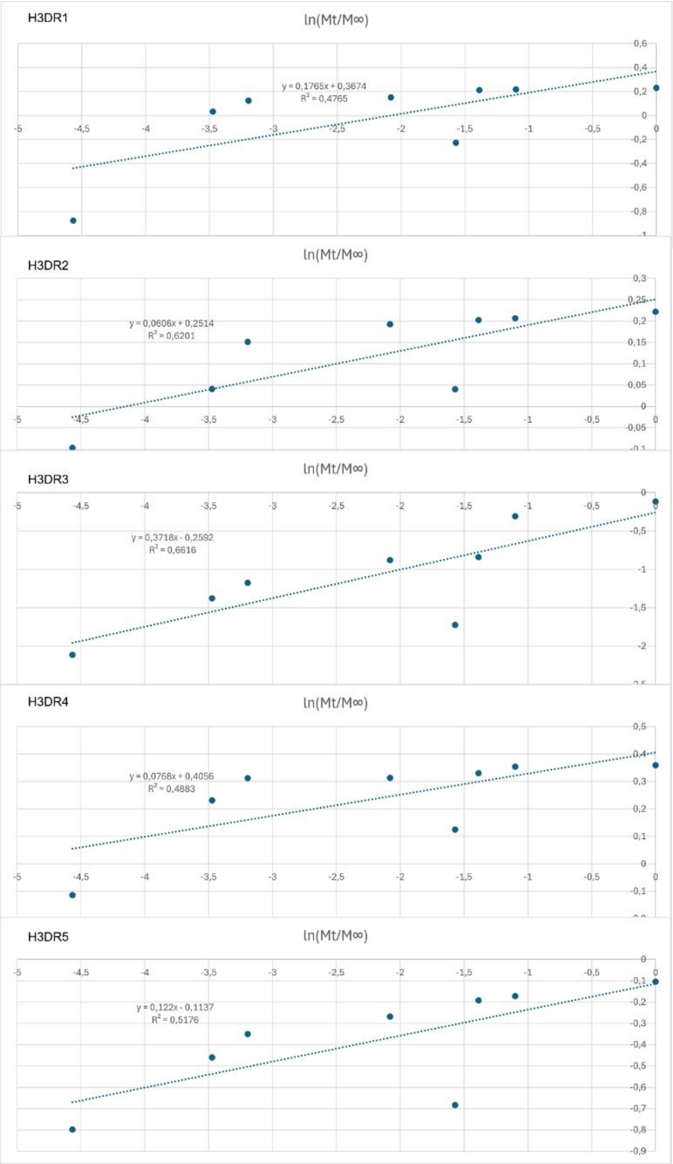

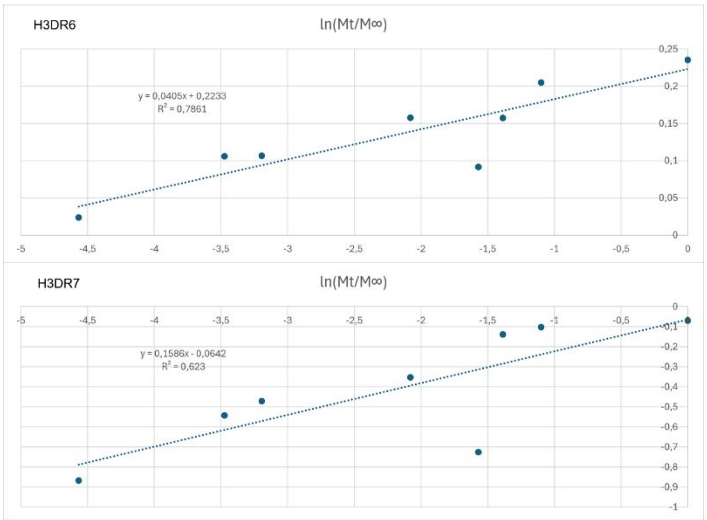

Figure S1. Non-linear fit of the experimental data using the Korsmeyer-Peppas model. The curve represents the best fit obtained by the model, showing the release profile of the compound from the matrix studied. The points represent the experimental data (mean ± standard deviation).

Metabolic activity

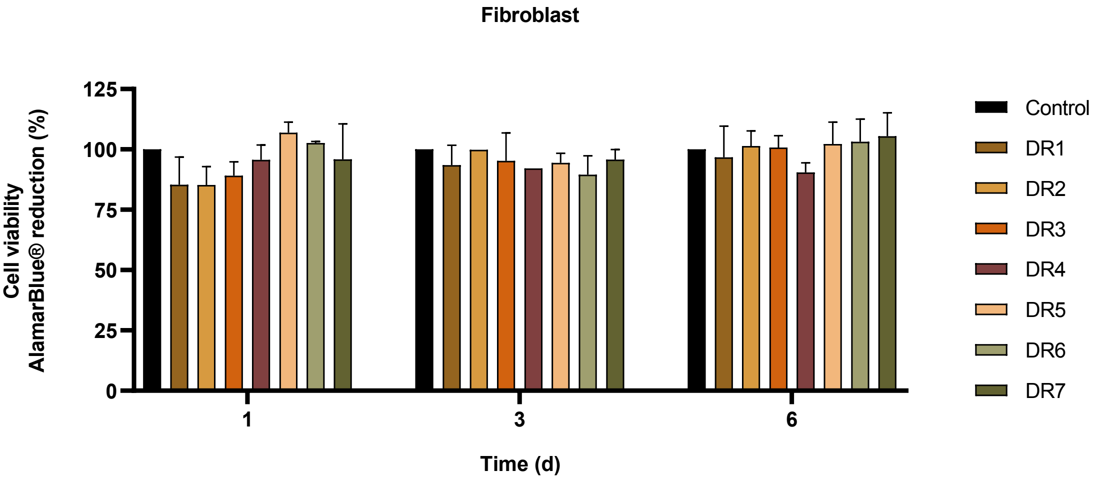

Figure S2. Metabolic activity of fibroblasts exposed to bioactive compounds derived from marine sponges *DR* over time. ANOVA and Tukey's post-hoc: Statistical differences represented by *. P<0,005.
